# Awareness of breast cancer risk factors and practice of breast self examination among high school students in Turkey

**DOI:** 10.1186/1471-2458-8-359

**Published:** 2008-10-17

**Authors:** Özgül Karayurt, Dilek Özmen, Aynur Çakmakçi Çetinkaya

**Affiliations:** 1 Department of Surgical Diseases Nursing, Dokuz Eylul University School of Nursing, Izmir, Turkey; 2 Department of Public Health Nursing, Celal Bayar University, School of Health, Manisa, Turkey

## Abstract

**Background:**

Young breast cancer patients have a lower rate of survival than old breast cancer patients due to being diagnosed at advanced stages. Breast self-examination makes women more "breast aware", which in turn may lead to an earlier diagnosis of breast cancer. The purpose of this study was to investigate knowledge and practice of breast self-examination and to determine knowledge of risk factors for breast cancer among high school students.

**Methods:**

This is a descriptive and cross-sectional study. It was conducted in a high school in Manisa, Turkey. The study sample included 718 female high school students. A socio-demographic characteristics data form, knowledge of breast self examination and risk factors for breast cancer form and breast self examination practice form were used to collect data.

**Results:**

The female high school students had insufficient knowledge about breast self-examination and a low percentage of students reported that they had performed breast self examination monthly. The most common reason for not doing breast self- examination was "not knowing how to perform breast self-examination" (98.5%). Most of the students had little knowledge of the risk factors for breast cancer. The most widely known risk factor by the students was personal history of breast cancer (68.7%). There was a significant relation between breast self-examination practice and age, school grade, knowledge about breast cancer and knowledge about breast self- examination.

**Conclusion:**

There is a need to increase knowledge of adolescent females about the risks of breast cancer and benefits of early detection. In fact, health care professionals can develop effective breast health care programs and help young women to acquire good health habits.

## Background

Cancer is the second leading cause of death in Turkey. Primary breast cancer is the most common cancer among Turkish women, which represents 26.5% of all female cancers [1]. Although breast cancer usually develops after the age of 45, the age of onset is decreasing, and more young women than ever are affected [2]. Young women's cancers are generally more aggressive and result in lower survival rates, making early detection even more important [3].

Promotion of self-care, an attitude fostered early in life, may pay lifelong dividends. The adolescent period is a time of rapid change that provides teaching opportunities for shaping health behaviors into adulthood. For example, teaching breast self-care may encourage positive behaviors such as performing breast self examination (BSE) and seeking regular professional breast examinations [4]. Health behaviors such as BSE can help empower women to take some control and responsibility over their health promotion [5]. For younger women, BSE education and adherence are a gateway to health promotion behaviors which set the stage for adherence to clinical breast examination and mammography screening later in life [3].

Although the value of BSE is controversial [6-8], American Cancer Society recommends as an option breast awareness and BSE for early detection of breast cancer. It benefits women in two ways: women become familiar with both the appearance and the feel of their breasts and detect any changes in their breasts as early as possible [9]. BSE makes women more "breast aware", which in turn may lead to an earlier diagnosis of breast cancer [10]. The rationale behind extending BSE practice as a screening test is the fact that breast cancer is frequently detected by women themselves without any other symptoms [11]. In Turkey, The Ministry of Health recommends BSE to increase awareness of breast cancer [12].

Although there were a lot of studies about knowledge of breast cancer and practice of BSE in female university students [5,13-15], there were few studies about the knowledge of breast cancer and practice of BSE in the age group of 14–19 years [4,16].

Studies from Turkey have revealed that 7.5% to 27% of university students performed BSE monthly [17,18]. There have not been any studies on high school students' knowledge about breast cancer and practice of BSE in Turkey.

In a study which evaluated the effectiveness of a BSE education program presented to ninth-grade girls, it has seen that girls who participated in the BSE education program recorded a significantly higher overall mean knowledge score than girls who did not participate. And also more girls who participated in the BSE education program reported performing BSE in the past month as well as a higher intention to perform BSE in the future [19].

The purpose of this study was to investigate knowledge and practice of BSE and to determine knowledge of risk factors for breast cancer among high school students in Turkey. The results of this study are of great importance in that obtained data about high school students' BSE knowledge and behaviors may assist nurses and other health professionals in planning health education programs for adolescent girls.

## Methods

### Design and Sample

This is a descriptive and cross-sectional study. The study population included high school students attending İsmet İnönü Anatolian Vocational High School for girls in Turkey in the 2006–2007 academic year (n = 886). The study sample included 718 students who were present in classes on the day when data were collected, who agreed to participate in the study and who filled in the questionnaires completely (RR% = 81%). İsmet İnönü Anatolian Vocational High School was located in Manisa, a city in the west part of Turkey.

### Ethical Considerations

Approval was obtained from the director of İsmet İnönü Anatolian Vocational High School. The students who were given information about the study and who accepted to participate in the study were included. Also, approval was obtained from the Directorate of School Education, affiliating with the Ministry of Education.

### Data Collection

Three instruments were used to collect data: a socio-demographic characteristics data form, knowledge of BSE and risk factors for breast cancer form and BSE practice form. Completion of the instruments took an average of 10 minutes.

### Instruments

#### Socio-demographic characteristics data form

Socio-demographic data elicited from students included their age, school grade, perceived income level, personal history of breast cancer, family history of breast cancer, whether they heard about BSE, information about breast cancer and BSE and source of information about breast cancer and BSE.

It was difficult to identify income level in Turkey because of a high inflation rate. Therefore, perceived income level was based on the participants' responses reflecting how they perceived their monthly income level. It was coded as 1 (income less than expenses), 2 (income equal to expenses) and 3 (income higher than expenses).

#### Knowledge of BSE and risk factors for breast cancer form

Knowledge of BSE was assessed with four multiple choice questions that included appropriate age to start BSE, knowledge about frequency of BSE, knowledge about appropriate time for BSE and knowledge of BSE procedure. The results of the studies by Maurer (1997) and Rashidi and Rajaram (2000) were taken into consideration when preparing these questions [20,21].

Knowledge of risk factors for breast cancer was assessed with 16 questions. The answers were "true", "false" and "don't know". This part assessed the presence of breast cancer risk factors with reference to the guidelines of the American Cancer Society (2007): 1. Family history of breast cancer; 2. Personal history of breast cancer; 3. Early menarche (< 12 years); 4. Late menopause (> 55 years); 5. Aging; 6. Alcohol; 7. Late age at first full-term pregnancy (> 30 years); 8. Never breastfed a child; 9. Recent oral contraceptive use; 10. Environmental pollution; 11. High fat diet; 12. Tobacco smoke; 13. Obesity (postmenopausal); 14. Recent and long-term use of hormone replacement therapy; 15. High-dose radiation to chest; 16. Lack of physical activity [9]. Cronbach alpha coefficient for the internal consistency reliability of this form was 0.80.

#### BSE practice form

The form was composed of three questions. The participants were asked whether they performed BSE and if they answered "yes", they were asked how often they performed BSE. Depending on the frequency of BSE, the participants were categorized as regular (the students who performed BSE every month) and not regular (those who performed BSE sometimes). Also, reasons for not doing BSE were assessed with one question.

### Data Analyses

Data were analyzed with SPSS v 10.0 and descriptive statistics. Chi-square analysis was made to determine the relation between BSE practice and age, school year, perceived income level and family history of breast cancer.

## Results

Participants in this study ranged in age from 14 to 19 years with a mean of 16.0 years (SD = 0.9). Of 718 students included in the study, 297 (41.4%) were ninth-grade students, 249 (34.6%) were tenth-grade students and 172 (24.0%) were eleventh-grade students. Twenty five percent of (n = 179) the participants had an income less than their expenses, 64.9% (n = 451) an income equal to their expenses and 10.2% (n = 73) an income higher than their expenses.

Seven percent (n = 52) of the students had a family history of breast cancer. None of the students had a personal history of breast cancer. More than half of the students (62.1%) reported that they had not heard about BSE. Thirty percent (n = 216) of the participants received information about breast cancer and BSE. Media were identified as the main source of information on breast cancer by 48.6% of the participants. Health professionals were mentioned as a source of information by 44.4% of the sample. Table 1.

**Table 1:** Characteristics of the sample (n = 718)

**Characteristics**	**n (%)**
**Age (Mean ± SD)**	16.0 ± 0.9
**Age**	
14–15	235 (32.7)
16–17	427 (59.4)
18–19	56 (7.9)
**School Grade**	
Ninth Grade	297 (41.4)
Tenth Grade	249 (34.6)
Eleventh Grade	172 (24.0)
**Perceived income level**	
Income less than expenses	179 (24.9)
Income equal to expenses	466 (64.9)
Income higher than expenses	73 (10.2)
**Personal history of breast cancer**	
Yes	--
No	718 (100.0)
**Family history of breast cancer.**	
Yes	52 (7.2)
No	666 (92.8)
**Heard about BSE**	
Yes	272 (37.9)
No	446 (62.1)
	
**Information about breast cancer and BSE**	
Yes	216 (30.1)
No	502 (69.9)
Source of information about breast cancer and BSE*	
Media	105 (48.6)
Health professionals	96 (44.4)
Book or journal	84 (38.9)
Other	50 (23.1)

* More than one choice was indicated for the question

Sixty-six percent (n = 480) of the students did not have knowledge about frequency of BSE, 75.4% (n = 542) of the students did not have knowledge about appropriate time for BSE and 65.4% (n = 469) of the students did not have knowledge of BSE procedure. Table 2.

**Table 2:** Students' knowledge of BSE

**Knowledge of BSE**	**n (%)**
**Knowledge about frequency of BSE**	
Don't know	480 (66.9)
Correct response	156 (21.8)
Incorrect response	82 (11.3)
**Knowledge about appropriate time for BSE**	
Don't know	542 (75.4)
Correct response	94 (13.2)
Incorrect response	82 (11.4)
**Knowledge of BSE procedure**	
Don't know	469 (65.4)
Correct response	191 (26.6)
Incorrect response	58 (8.0)

The percentage of the students who performed BSE regularly every month was 6.7%. Twenty percent of the students reported that they performed BSE irregularly. The most common reasons for not doing BSE were "not knowing how to perform BSE" (98.5%), "not expecting to get breast cancer" (45.6%) and "not having a close relative with breast cancer" (42.9%). Table 3.

**Table 3:** Students' BSE performance

**BSE performance and reasons for not performing BSE**	**n (%)**
**BSE performance**	
No	524 (73.0)
Irregular	181 (20.3)
Regular	13 (6.7)
Reasons for not doing BSE (524)*	
Not knowing the frequency of BSE	181 (35.0)
Not knowing how to perform BSE	516 (98.5)
Not having time	52 (10.1)
Not expecting to get breast cancer	239 (45.6)
Not giving importance to health	35 (6.8)
Having more important problems	12 (2.3)
Fear of finding a breast lump	44 (8.5)
Not having a close relative with breast cancer	222 (42.9)

*More than one choice was indicated for the question

Most of the students had little knowledge of the risk factors for breast cancer. The most widely known risk factors by the students were personal history of breast cancer (68.7%) and family history of breast cancer (67.0%). Recent oral contraceptive use and late menopause were not known as a risk factor for breast cancer by most of the students. Table 4.

**Table 4:** Students' knowledge of risk factors for breast cancer

**Risk Factors for Breast Cancer**	**True**		**False**		**Don't know**	
	
	**N**	**%**	**N**	**%**	**N**	**%**
Obesity (postmenopausal)	168	23.4	124	17.3	426	59.3
Family history of breast cancer	481	67.0	65	9.0	172	24.0
High fat diet	274	38.2	77	10.7	367	51.1
Early menarche (< 12 years)	59	8.2	172	24.0	487	67.8
Late menopause (> 55 years)	82	11.4	96	13.4	540	75.2
Tobacco smoke	456	63.5	57	8.0	205	28.5
Alcohol	416	57.9	55	7.7	247	34.4
Aging	164	22.8	179	24.9	375	52.3
Environmental pollution	280	39.0	104	14.5	334	46.5
Personal history of breast cancer	493	68.7	24	3.3	201	28.0
Late age at first full-term pregnancy (> 30 years)	149	20.8	140	19.5	429	59.7
Never breastfed a child	193	26.9	135	18.8	390	54.3
Recent oral contraceptive use	51	7.1	120	16.7	547	76.2
Recent and long-term use of hormone replacement therapy	148	20.6	42	5.8	528	73.6
High-dose radiation to chest	338	47.1	34	4.7	346	48.2
Deficiency of physical activity	311	43.3	85	11.9	322	44.8

The relationship between BSE practice and age, school year, perceived income level and family history of breast cancer, information about breast cancer and BSE is shown in. Table 5. Chi-square analysis showed that there was a significant relation between BSE practice and age, school year, information about breast cancer and BSE. There was no significant relation between BSE practice and perceived income level and family history of breast cancer.

**Table 5:** The relations between socio-demographic variables and BSE practice (n = 718)

**Selected Variables**	**Yes** **N (%)**	**No** **N (%)**	**X**^2^	**P**
**Age (years)**				
14–15	50 (21.3)	185 (78.7)		
16–17	129 (30.2)	298 (69.8)	6.137	0.046
18–19	15 (26.8)	41 (73.2)		
**School Grade**				
Ninth-grade	64 (21.5)	233 (78.5)		
Tenth-grade	78 (31.3)	171 (68.7)	7.749	0.021
Eleventh-grade	52 (30.2)	120 (69.8)		
**Perceived income level**				
Income less than expenses	41 (22.9)	138 (77.1)		
Income equal to expenses	128 (27.5)	338 (72.5)	3.518	0.172
Income higher than expenses	25 (34.2)	48 (65.8)		
**Family history of breast cancer**				
Yes	20 (38.5)	32 (61.5)	3.722	0.054
No	174 (26.1)	492 (73.9)		
**Information about breast cancer and BSE**				
Yes	95 (44.0)	121(56.0)	45.076	0.001
No	99(19.7)	403(80.3)		

## Discussion

Developing proper health habits in adolescence should lead to maintenance of good health in adulthood. These habits can have profound, long-term ramifications on health. One of these habits is breast self examination [22]. To our knowledge, there have been no studies about this issue in Turkey and there have been only a few studies about the issue in adolescents in the world. Therefore, the results of this study could not be compared widely. This study provided important data about breast health awareness of high school students in Turkey. In fact, adolescent females had poor knowledge of breast cancer and BSE.

### Socio-demographic features associated with BSE

Only 37.9% of the students reported to hear about BSE and 30.1% of students had acquired information about breast cancer and BSE. Nearly half of the students reported their main source of information on breast cancer and BSE as media, consistent with the results of the studies by Budden (1995) and Milaat (2000) [5,16]. These findings indicated that media continued to be one of the most important sources of information about breast cancer and BSE and highlighted the cooperation between public health educators and the media in dissemination of breast cancer information and BSE.

### Knowledge of BSE

This study showed that adolescent females in Turkey may not have sufficient knowledge about BSE. In fact, a small percentage of the students had knowledge about appropriate time for BSE (13.2%), frequency of BSE (21.8%), and BSE procedure (26.6%). In a study by Budden, 77% of the female students correctly identified the recommended time for BSE [5], but only 13% of the first year of nursing students did so [15]. Milaat reported that 14.4% of secondary-school female nursing students had knowledge about the frequency of BSE and that 7.1% of the students had knowledge about appropriate time for BSE [16]. Ludwick and Gaczkowki (2001) found that 55.7% of teenagers did not know how to perform BSE [4]. In a study conducted in Turkey Beydag and Karaoglan (2007) found that 50% of female university students did not know how to perform BSE [18]. These findings have suggested that adolescents know less about BSE than older groups. The Young Survival Coalition pointed out that BSE was the only method of early detection of breast cancer for young women [23]. In their research, 83% of young women reported to have found the lump themselves [3]. It is important for younger women to become familiar with how their breasts look and feel through monthly BSE [2,3,24].

### BSE Practice

In this study, 6.7% of the students were performing BSE monthly and 20.3% of the students were performing BSE irregularly. Students' knowledge about BSE might have affected their monthly BSE performance. However, only a small number of students who had knowledge about the BSE procedure were performing BSE monthly. In other studies the percentage of monthly BSE performance have been found to be 3.4% among teenagers [4], 14.8% among students aged 17 to 30 years in Europe [25], 37% among female university nursing students in Australia [5], and 27% among female nursing students [14]. These studies suggest that the percentage of adolescents performing BSE monthly is low all over the world. Studies from Turkey have revealed that the percentage of older women performing BSE monthly ranged from 10.2% to 13% [26,27]. It is expected that a higher percentage of older women perform BSE because they are at higher risk of breast cancer. However, studies from Turkey have shown that low percentages of both young and older women perform BSE. It may be that education programs organized to increase breast health awareness are not sufficient. Such education programs should start in early years of life.

### Reasons for not doing BSE

The most common reasons for not doing BSE were "not knowing how to perform BSE" (98.5%), "not expecting to get breast cancer" (45.6%) and "not having a close relative with breast cancer" (42.9%). Many people believe that they do not get cancer in adolescence. Adolescents tend to deny that they are also vulnerable to disease like all humans. Lack of knowledge about how to perform BSE among Turkish adolescents might have been due to insufficient education programs for breast health awareness. Breast health awareness provides women with some acknowledgement of the part they can play in being empowered to fight breast disease [28].

Consistent with the results of this study, in many studies, students noted that they did not perform BSE because they did not know how to perform it [4,29], and that they did not have a family history of breast cancer [30].

### Breast cancer risk factors

The results showed that most of the students had little knowledge of the breast cancer risk factors. The most widely known risk factors by the students were personal history of breast cancer (68.7%) and family history of breast cancer (67.0%). In other words, students were aware that breast cancer was associated with genetic factors, which has a positive effect on breast health among young women. This increased awareness in the role of genetic factors in breast cancer, which can be attributed to extensive coverage of this risk factor in Turkish media.

The idea that lifestyle changes may modify the risk of developing breast cancer is supported by several lines of evidence. In fact, McTiernan (2003) reported that lifestyle changes may be expected to have an impact on young women and help them to avoid the risk factors [31]. In this study, the students turned out to know little about lifestyle changes to correct breast cancer risk factors such as obesity, high fat diet, smoking, and alcohol use. These breast cancer risk factors can be changed with health education. So health care professionals can play an important role in educating students, enhance their awareness in breast cancer risk factors and influence their behavior.

### The relations between socio-demographic variables and BSE practice

In the present study there was a significant relation between BSE practice and age and school grade, information about breast cancer and BSE, but there was no significant relation between BSE practice and perceived income level and family history of breast cancer. Alsaif (2004) claimed that women who anticipated favorable outcomes in general were more confident in their breast and revealed a relation between BSE practice and school grade [13], and between BSE practice and age [13,32,33]. There have been studies with conflicting results as well. While two studies showed a relation between family history of breast cancer and regular BSE performance [34,35], another two studies revealed no relation between family history of breast cancer and BSE performance [5,32].

### The Limitations of Study

Since the sample of this study included students of a high school, the results of the study cannot be generalized to the larger population in Turkey.

## Conclusion

Health behaviors that are formed during adolescence can enhance future health and have implications for the entire life course. Female high school students had little knowledge of risk factors for breast cancer. Students were also not familiar with BSE. The results of this study may provide important baseline information about awareness of breast cancer risk factors and practice of breast self-examination among high school students. There is a need to increase knowledge of adolescent females about the risks of breast cancer and benefits of early detection. Health care professionals should develop effective breast health programs in adolescence to help adolescent females acquire good health habits from their youth.

## Abbreviations

BSE: Breast self-examination.

## Competing interests

The authors declare that they have no competing interests.

## Authors' contributions

ÖK and DÖ were responsible for the study conception and design and drafting of the manuscript. AÇ and DÖ performed the data collection, DÖ and AÇ conducted the data analysis. ÖK and DÖ made critical revisions of the paper. All authors read and approved the final manuscript.

## Pre-publication history

The pre-publication history for this paper can be accessed here:


